# The effects of fresh *Gastrodia elata* Blume on the cognitive deficits induced by chronic restraint stress

**DOI:** 10.3389/fphar.2022.890330

**Published:** 2022-08-29

**Authors:** Hong Huang, Yiwen Zhang, Caihong Yao, Qinghu He, Fang Chen, Han Yu, Guanghua Lu, Ning Jiang, Xinmin Liu

**Affiliations:** ^1^ Research Center for Pharmacology and Toxicology, Institute of Medicinal Plant Development (IMPLAD), Chinese Academy of Medical Sciences and Peking Union Medical College, Beijing, China; ^2^ Sino-Pakistan Center on Traditional Chinese Medicine, Hunan University of Medicine, Huaihua, China; ^3^ Hunan University of Chinese Medicine, College of Traditional Chinese Medicine, Changsha, China; ^4^ School of Pharmacy, Chengdu University of Traditional Chinese Medicine, Chengdu, China; ^5^ Institute of Drug Discovery Technology, Ningbo University, Ningbo, China

**Keywords:** chronic restraint stress, *Gastrodia elata* Blume, learning and memory, animal behavior, mitochondria

## Abstract

Chronic restraint stress (CRS) is a classic animal model of stress that can lead to various physiological and psychological dysfunctions, including systemic neuroinflammation and memory deficits. Fresh *Gastrodia elata* Blume (FG), the unprocessed raw tuber of *Gastrodia elata* Blume, has been reported to alleviate the symptoms of headache, convulsions, and neurodegenerative diseases, while the protective effects of FG on CRS-induced cognitive deficits remain unclear. This work aimed to evaluate the effects of FG on CRS-induced cognitive deficits through multiplex animal behavior tests and to further explore the related mechanism by observing the expression of mitochondrial apoptosis-related proteins in the mouse hippocampus. In *in vivo* experiments, mice were subjected to the object location recognition test (OLRT), new object recognition test (NORT), Morris water maze test (MWMT), and passive avoidance test (PAT) to evaluate the learning and memory ability. In *in vitro* experiments, the expression of the AKT/CREB pathway, the fission- and apoptosis-related proteins (Drp1, Cyt C, and BAX), and the proinflammatory cytokines’ (TNF‐α and IL‐1β) level in the hippocampus was examined. Our results demonstrated that in spontaneous behavior experiments, FG significantly improved the cognitive performance of CRS model mice in OLRT (*p* < 0.05) and NORT (*p* < 0.05). In punitive behavior experiments, FG shortened the escape latency in long-term spatial memory test (MWMT, *p* < 0.01) and prolonged the latency into the dark chamber in non-spatial memory test (PAT, *p* < 0.01). Biochemical analysis showed that FG treatment significantly suppressed CRS‐induced Cyt C, Drp1, and BAX activation (*p* < 0.001, *p* < 0.01 and *p* < 0.05), promoted the CREB, p-CREB, AKT, and p-AKT level (*p* < 0.05, *p* < 0.01 and *p* < 0.001), and inhibited the CRS‐induced proinflammatory cytokines (TNF‐α and IL‐1β, *p* < 0.05 and *p* < 0.001) level in the hippocampus. Taken together, these results suggested that FG could attenuate cognitive deficits induced by CRS on multiple learning and memory behavioral tests.

## 1 Introduction

Stress is a common nonspecific response which results when individuals feel threatened, physically or mentally. Modern scientific research has demonstrated a high correlation between social and/or physical stress and memory impairment (Bowman et al., 2003). A chronic restraint stress (CRS) model simulates the physiological and psychological stress of humans after long-term exposure to confined spaces by placing animals in confined spaces and restricting their behavior and activities, which leads to depression, anxiety, learning, and memory deficits (Huang et al., 2015; Woo et al., 2018). Numerous research studies have shown that CRS diminished the learning and memory ability of animals (Kezhu et al., 2017; Dong et al., 2019). Studies have revealed that CRS activates glial cells to release proinflammatory factors, such as interleukin-1β (IL-1β) and tumor necrosis factor α (TNF- α) (Deak et al., 2015), which in turn promotes apoptosis, ultimately leading to apoptosis. BAX is a proapoptotic protein belonging to the Bcl-2 family, and it acts in conjunction with Drp1 to increase mitochondrial membrane permeability and division, which facilitates the release of cytochrome c (Cyt C) from the mitochondria into the cytoplasm (Reed, 1997; Sugioka et al., 2004; Hu et al., 2020). CREB serves an important role in neuronal regeneration, synaptic growth, learning, and memory. AKT also plays a major role in promoting neuronal survival and synaptic plasticity (Wang et al., 2020a). The activation of the AKT/CREB pathway provides direct links to neuroprotective mechanisms (Zhang et al., 2020).

The well-known herb plant fresh *Gastrodia elata* Blume (FG) has been commonly used as dietary supplement and medicine. The traditional function of FG is known for carminative, which is primarily used to treat headache, dizziness, and rheumatic ache (Zhan et al., 2016). Modern pharmacological studies have demonstrated that it is mainly effective on the central nervous system and cardiovascular system (Dai et al., 2017; Liu et al., 2018) and has a good effect on neuropsychiatric diseases such as insomnia, Alzheimer’s disease, depression, and anxiety disorder (Jung et al., 2006; Zhang et al., 2012; Huang et al., 2013; Huang et al., 2021a). These effects may be due to its anti-inflammatory, antiapoptotic, and promoting neurogenesis (Hsu et al., 2021; Wang et al., 2022). Our previous study has shown that FG possessed good function with improving learning and memory impairment caused by insufficient sleep (Huang et al., 2021b). However, the protective effects of FG on CRS-induced cognitive deficits are rarely investigated.

In this study, we researched the effects of FG on CRS-induced cognitive deficits through multiplex animal behavior tests. The possible neuroprotection mechanisms of FG were investigated through the modulation of inflammation and the AKT/CREB pathway in the hippocampus after CRS. Our research aims to address the effect of FG on the cognitive deficits induced by CRS and provides evidence for the application of FG in ameliorating stress-induced hypomnesia.

## 2 Methods

### 2.1 Animals

120 ICR mice weighing between 20 and 23 g were purchased from the Vital River Co., Ltd. (Qualified No. SCXK 2021–0006, Beijing, China). All experimental protocols adhered to the guidelines of the Animal Care and Use Committee of the Institute of Medical Plant Development, Chinese Academy of Medical Sciences, and Peking Union Medical College (Permit Number: 2020122411505). The animals were housed under standard laboratory conditions: room temperature was 24°C ± 2°C, relative humidity was 55°C ± 10°C, and maintained on a 12 h light/dark cycle (lights on at 8:00 a.m.). The animals arrived at least 3 days before the experiments and were handled carefully.

### 2.2 Chemicals and reagents

Donepezil hydrochloride (DNP [Aricept], Eisai Inc. [Ibaraki, Japan]) was used as a positive control. TNF-α (lot No. 040621210429) and IL-1β (lot No. 040921210429) commercial kits were obtained from Beyotime (Shanghai, China). A detailed description for primary antibodies used in the present research is displayed in Table 1, and secondary antibody is goat anti-rabbit IgG-HRP (Gene-Protein Link, lot No.07001). Reference substances: gastrodin (lot No. 110807–201507, purity ≥ 98%) was purchased from the China Institute for Food and Drug Control; p-hydroxybenzyl alcohol (lot No. H20806, purity ≥ 98%) was purchased from Sigma-Aldrich company; and parishin E (lot No. 1–1011606) was purchased from Chengdu Purifa Technology Development Co., Ltd. (Chengdu, China).

**TABLE 1:** Antibodies used in the present study.

Primary antibodies	Source	Dilution	Molecular weight/kDa	Origin
AKT	Rabbit	1:1000/WB	60	CST (#4685)
BAX	Rabbit	1:2000/WB	21	Abcam (#ab32503)
CREB	Rabbit	1:1000/WB	37 (40)	Abcam (#ab32515)
Cyt C	Rabbit	1:5000/WB	11	Abcam (#ab133504)
Drp1	Rabbit	1:1000/WB	83	Abcam (#ab184247)
GAPDH	Rabbit	1:2000/WB	36	ABClonal (#A19056)

The equipment used included an open‐field computer‐aided controlling system (KSYY-OP- V4.0), OLRT and NORT system (KSYY-OR- V2.0), PAT system (KSYY-AD-V4.0), and MWMT apparatus (KSYY-MWM-V4.0), which were jointly developed by Beijing Kangsen High-technology Co., Ltd., China Astronaut Research and Training Center, the Institute of Medicinal Plant Development, and Chinese Academy of Medical Sciences. Ultra-high-performance liquid chromatography was performed (UPLC, Thermo Scientific Ultimate 3000, United States).

### 2.3 The preparation and standardization of fresh *Gastrodia elata* Blume

#### 2.3.1 Sample prepared

Fresh *Gastrodia elata* Blume tuber was purchased from the local herbal grower in Jinkouhe, Sichuan Province, China. The specimen was identified as a tuber of *Gastrodia elata* Blume by Guang-hua Lu, professor at Chengdu University of Traditional Chinese Medicine, Chengdu, China.

To obtain FG, the fresh *Gastrodia elata* was pureed and distilled water was added 2–3 times in a liquidizer until completely smooth. Then, the puree was filtered with gauze, the filter residue was taken, and distilled water was added; the procedure was repeated once to ensure the complete extraction of the content. Then, the extract was filtered, freeze-dried, and stored at 4°C until utilization.

#### 2.3.2 Fingerprint of fresh *Gastrodia elata* Blume

The FG (0.2 g) was dissolved in 60% ethanol (25 ml); ultrasound extraction was performed for 45 min followed by filtration through a 0.45 μm membrane filter, and the subsequent filtrate was gathered for testing. The sample was performed on an UPLC system with a Accucore Vanquish C_18+_ column (100 mm × 2.1 mm × 1.5 μm, Thermo Scientific). The column was operated at 35°C. The gradient elution consisted of a mobile phase of (A) 0.075% formic acid acetonitrile and (B) 0.1% formic acid water using a gradient elution of 2 %–5% A at 0–4 min, 5%–14% A at 4–10 min, 14%–53% A at 10–18 min, and 53%–95% A at 18–20 min. The flow rate was maintained at 0.4 ml/min, and aliquots of 1 μL were injected into the UPLC system, and the detection wavelength was set at 220 nm.

**FIGURE 1:**
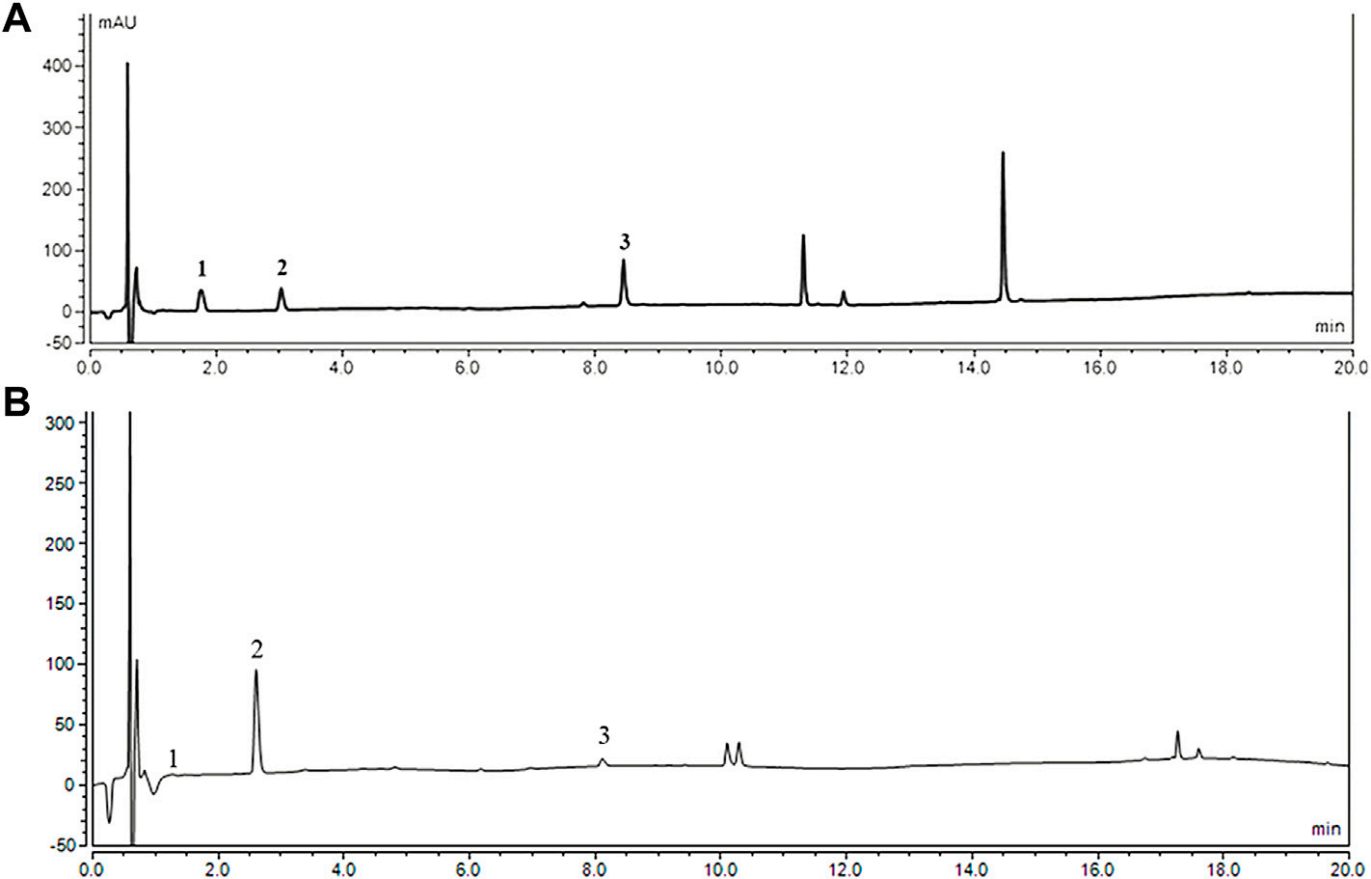
HPLC chromatograms of reference substance **(A)** and sample of FG. **(B)** (1) gastrodin; (2) p-hydroxybenzyl alcohol; and (3) Parishin E.

The content of gastrodin, p-hydroxybenzyl alcohol, and parishin E in the FG extract detected gastrodin, p-hydroxybenzyl alcohol, and parishin E was 0.264, 6.485, and 2.012 mg/g, respectively (shown in Figure 1).

### 2.4 Groups and drug treatments

The mice were divided into five groups pseudorandomly (*n* = 12 in each group) as follows: the control group, CRS model group, CRS + DNP treatment group (i.g., 1.6 mg/kg), the dose is according to literature (Yang et al., 2020), and CRS + FG treatment groups (i.g., 0.5 and 1 g/kg, equivalent to the crude material), the dosage was based on our preliminary studies and literature (Lin et al., 2018; Huang et al., 2021b). The FG and DNP were dissolved in distilled water; mice in the control and CRS model group were administrated with dissolvent and treated with corresponding drugs from the 21st of CRS until sacrifice.

### 2.5 The chronic restraint stress procedure

**FIGURE 2:**
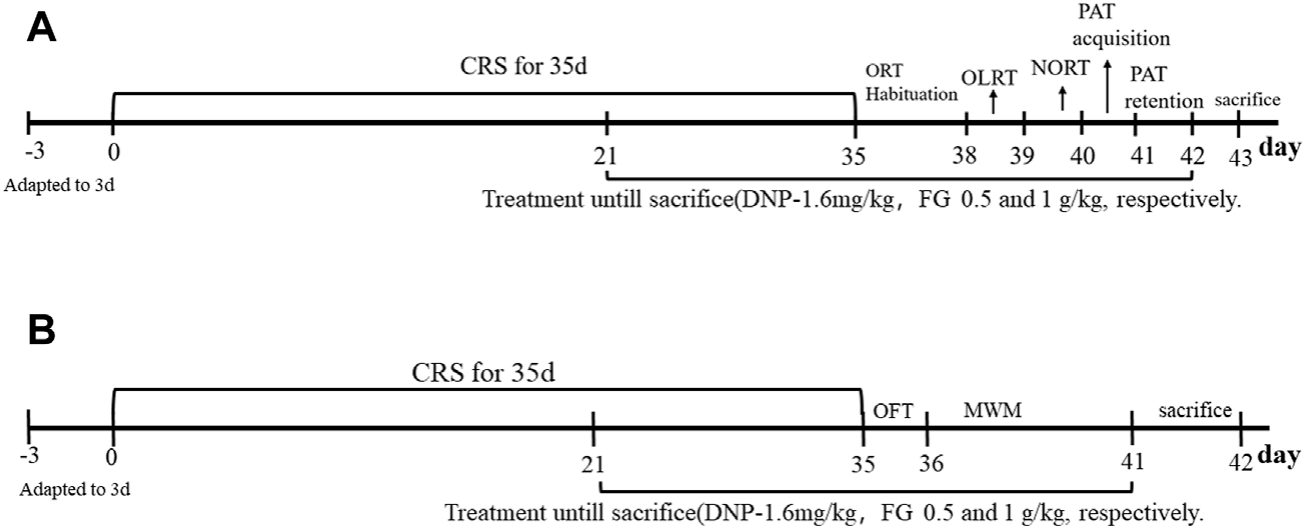
Experimental timeline of the study. **(A)**, **(B)** Two experiments.

The animals were subjected to CRS as described in the prior study (Wang et al., 2020b). During the CRS period, the mice were kept in a transparent Plexiglas restrainer (L 13 × D 4 cm) with air holes and fixing plug for 10 h (from 22:00 p.m. to 8:00 a.m.) each day for 35 consecutive days. After stress, the mice returned to their cages under *ad libitum* access to food and water. The control mice were fasted and deprived of water during the CRS process. The experimental procedure is shown in Figure 2. Body weight was measured every 7 days during the experiment to monitor the effects of CRS and drug treatment on mice.

### 2.6 Behavioral tests

#### 2.6.1 Open-field test

The open-field test (OFT) is used to evaluate the locomotor activity and sedative/excitatory states of animals. In neuropsychiatric behavioral experiments, when the locomotor activities of animals in each group have no significant difference, the next animal behavior tests have the significance of statistics (O’Connor et al., 2003).

The instrument consists of an infrared camera which is directly above the center of four circular test boxes (H 28 × D 28 cm). The drugs were administered 30 min before the experiment. Each animal was placed into the center of the tank in a proper sequence and allowed to explore freely for 3 min. The total distance and average speed in the next 10 min were recorded automatically by open-field computer-aided controlling system and as an index of locomotor activity.

#### 2.6.2 Object recognition test

This sensitive cognitive behavior detection method allows to measure rodents’ instinct to approach and explore novel objects. The detection function of the object recognition test varies according to different experimental requirements, but the most commonly used are object location recognition test (OLRT) and new object recognition test (NORT).

#### 2.6.3 Object location recognition test

The equipment and procedure of OLRT were consistent and described previously (Lu et al., 2018a). A 3-day habituation period of the OLRT was initiated. In this phase, the mice were exposed to an empty arena and allowed to explore for 10 min. The experiment test phase consisted of two parts: the familiar phase and the test phase. During the familiar phase, the mice were allowed to explore the arena for 5 min, containing two identical objects in a parallel position on the same side. After 30 min interval, the mice returned to the arena for 5 min, with one of the original objects stationed at the same position (Familiar, F) and another one placed diagonally referred as new location (Novelty, N). To avoid biases, locations for the objects were counterbalanced among the groups. In each step, the floor of the arena and objects were cleaned with 75% ethanol to avoid odor interference. The learning and memory abilities of animals in this test were evaluated by the discrimination index (DI).

$$DI=\left(T_{N}-T_{F}\right)/\left(T_{N}+T_{F}\right),$$

where T_N_ and T_F_ indicate the time of exploring new and familiar location within 5 min, respectively.

#### 2.6.4 New object recognition test

The familiar conduct of NORT is same as OLRT, whereas for the test phase, one object of the original objects was replaced by a new matching one (Novel, N) and another object was retained (Familiar, F). The learning and memory abilities of animals in this test were also evaluated by the discrimination index (DI).

$$DI=\left(T_{N}-T_{F}\right)/\left(T_{N}+T_{F}\right),$$

where T_N_ and T_F_ indicate the time of exploring new and familiar object within 5 min, respectively.

#### 2.6.5 Morris water maze test

The Morris water maze test (MWMT) is a classic experiment to evaluate the acquisition of animal spatial memory. In MWMT, the experimental animals are forced to swim to find the platform hidden in water to escape through repeated trials and reference distal cues (Tian et al., 2019). The animals’ spatial learning and memory ability can be effectively reflected by this method, which includes acquisition, maintenance, and reproduction (Lu et al., 2020). The MWMT device includes a circular pool (100 cm in diameter and 38 cm in height) filled with water (24–26°C) turbid by adding ink and divided into four quadrants. A cylindrical platform is placed in one quadrant (e.g., the first quadrant) and 1–1.5 cm below the water surface. There is a camera above for monitoring. The experiment was for five consecutive days and received training twice a day. Each time, the animals were introduced (facing the pool wall) into each of the four quadrants in turn (except for target quadrant), then they were given 90 s to find the platform, and the mice were placed on the platform for 10 s before and after entering the water. The escape latency (time of found the platform), escape success rate, and other indicators were recorded by the computerized tracking and image analyzer system.

#### 2.6.6 Passive avoidance test

The passive avoidance test (PAT) was performed as described by previous the literature (Xu et al., 2017). The apparatus in trough-shape consists of a white illuminated chamber and a dark chamber (L 17 cm × W 13.5 cm × H 25 cm) connected by a small arch door. In acquisition trial, each mouse was placed into the light chamber for adapting for 180 s, followed by turning on the current for 300 s. When it entered the dark chamber, a 0.5 mA electric foot shock (5 s) was delivered. After 24 h, the retention trial was performed again as the acquisition trial without adaptation period. The mice were placed into the light chamber without adapting, and the latency (the first-time mice enter the dark chamber) was recorded in 300 s test session. If the mice did not enter the darkroom within 300 s, recorded as 300 s. Normal animals prefer to enter and stay in the dark room, but once entering or staying in the dark room, they receive electric shock. After several repetitions, they finally learn that the brighter room is the safety area to avoid electric injury (Xu et al., 2016a; Lu et al., 2018b).

### 2.7 Preparation of brain tissues

The animals were sacrificed after the behavioral tests, the hippocampus of which was dissected from the whole brain on ice immediately, snap-frozen, and stored at -80°C for further analysis.

### 2.8 Enzyme-linked immunosorbent assays

After the last behavioral test, the separated hippocampal from each hemisphere was weighed and homogenized in 0.9% saline (1:9, w: v). The levels of TNF-α and IL-1β were determined by commercial enzyme-linked immunosorbent assay (ELISA) kits according to the manufacture’s protocols from Beyotime (Shanghai, China).

### 2.9 Western blot assay

The hippocampus tissue samples were weighed and homogenized in lysis buffer (Solarbio, Beijing, China, lot: BC3710). After the homogenate was centrifuged at 12,000 rpm for 30 min at 4°C, the supernatant was collected and determined by a BCA protein assay kit (CWBIO, Beijing, China, lot: 32220). The equal amount of protein was separated by 10% SDS-PAGE gels (CWBIO, Beijing, China, lot: 01413), and then transferred onto polyvinylidene fluoride (PVDF) membrane (Millipore, MA, United States). The PVDF membrane was blocked by 5% skim milk in Tris-buffered saline and Tween 20 solution (TBST) at room temperature for 90 min. Subsequently, the membrane was incubated by appropriate primary antibodies overnight at 4°C. The membrane was washed in TBST three times for 10 min and incubated with corresponding HRP-conjugated secondary antibodies at room temperature for 90 min. After three washings for 10 min with TBST, the bands were detected by ECL kit (Absin, Shanghai, China, lot: OA16) and visualized by using a Molecular Imager ChemiDoc XRS + System (Bio-Rad, CA, United States).

### 2.10 Statistical analysis

Data were analyzed by SPSS software 21.0 (Chicago, IL, United States) and expressed as the mean ± standard error of the mean (Mean ± SEM). The exclusion of outliers follows a standard analytical procedure to avoid undue influence. Differences among the experimental groups were determined by one-way analysis of variance (ANOVA) followed by least significant difference post hoc test. The data recorded from the acquisition trails of the MWMT among the groups over a period of 5 days were analyzed with repeated measures and a multivariate analysis of variance (ANOVA) process of the general linear model. *p* < 0.05 was considered statistically significant and *p* < 0.01 indicates a very significant difference.

## 3 Results

### 3.1 Effects of fresh *Gastrodia elata* Blume on the body weight and locomotor activity in chronic restraint stress-treated mice

**FIGURE 3:**
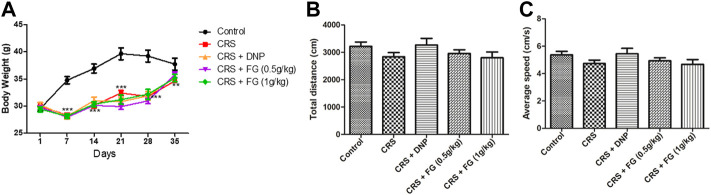
Effects of CRS, FG, and DNP on the locomotor activity of CRS mice in the open-field test and body weight. **(A)** Body weight; **(B)** total motion distance; and **(C)** average speed. Values represent the mean ± S.E.M. *n* = 10 ∼ 12 mice/group.

At the beginning of the experiment, there was no significant difference in body weight among the groups. Following 35 days of CRS, body weight of the CRS groups decreased significantly (*p* < 0.05) compared with the control group (Figure 3A). By the way, the CRS group showed grooming deficit after 5 days and this appearance defect remained until the end of the experiment.

As shown in Figure 3B, there was no significant changes among all groups in the total distance (F_4,52_ = 1.351, *p* > 0.05) and the average speed (F_4, 52_ = 1.346, *p* > 0.05), which indicated that CRS and drugs treatment did not affect the locomotor activities of mice.

### 3.2 Effects of fresh *Gastrodia elata* Blume on the object recognition test in chronic restraint stress-treated mice

**FIGURE 4:**
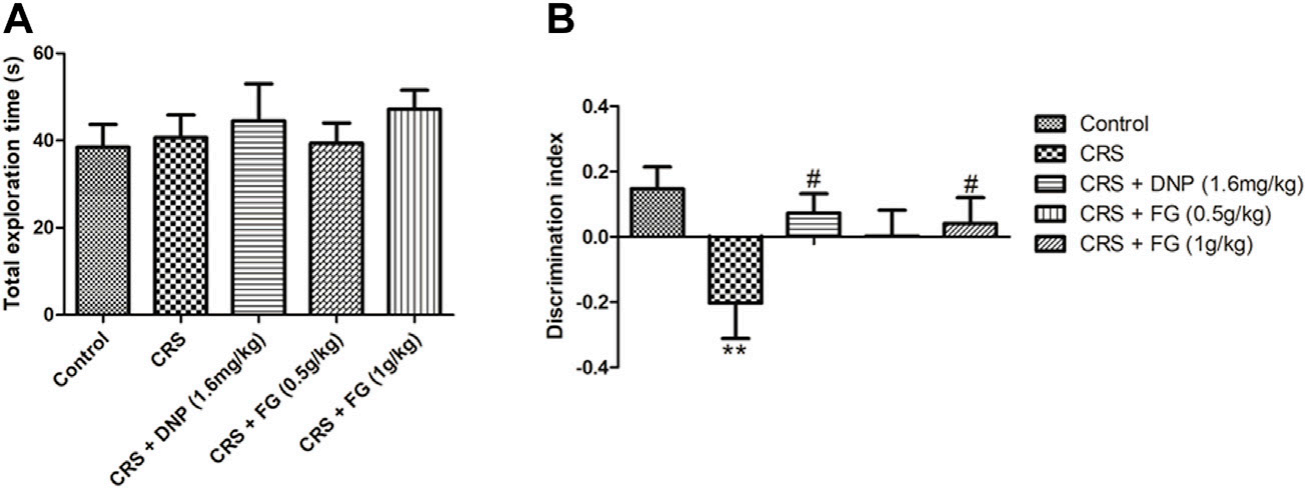
Effect of CRS, FG, and DNP on the object location recognition test of mice induced by CRS. **(A)** Total exploration time in familiar phase. **(B)** DI in test phase. Values represent the mean ± S.E.M. *n* = 8 ∼ 12 mice/group. **p < 0.01 compared with the control group; ^#^ *p* < 0.05 compared with the CRS group.

The effect of FG on spontaneity, spatial learning, and memory was evaluated by the OLRT. In a familiar phase, there was slight increase in drugs-treated groups in the total exploration time of the objects without significance differences among all the groups (F_4, 43_ = 0.772, *p* > 0.05, Figure 4A). The results guaranteed that there were no differences in the animals’ ability of exploration for the objects. In the test phase, as shown in Figure 4B, the DI of the CRS model group was decreased significantly (F_4, 44_ = 0.698, *p* < 0.01) compared with the control group, while the DNP and FG (1 g/kg) treatment groups elevated the discrimination index with significance (F_4, 44_ = 0.446, *p* < 0.05) compared with the CRS model group. It illustrated that these treatments could reverse the CRS-induced memory deficit.

**FIGURE 5:**
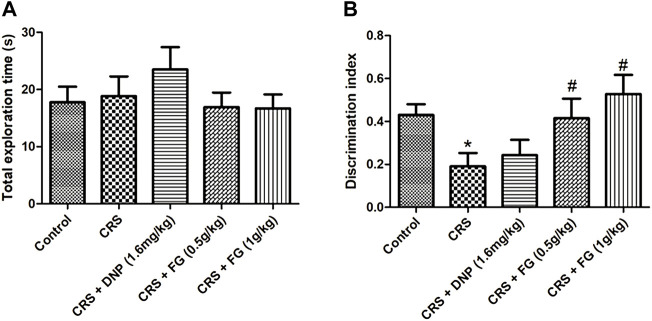
Effect of CRS, FG, and DNP on the new object recognition test of mice induced by CRS. **(A)** Total exploration time in familiar phase. **(B)** DI in test phase. Values represent the mean ± S.E.M. *n* = 8 ∼ 12 mice/group. ^*^ *p* < 0.05 compared with the control group; ^#^ *p* < 0.05 compared with the CRS group.

Moreover, NORT was used to evaluate the effects of FG on spontaneity non-spatial learning and memory in CRS-treated mice. Similarly, there was no significant difference among all groups in a familiar phase (F_4, 46_ = 0.704, *p* > 0.05, Figure 5A). In the test phase, the DI of the CRS model group was decreased significantly (*p* < 0.05) compared with the control group, while the FG (0.5 and 1 g/kg) treatment groups increased the discrimination index with significance (F_4, 40_ = 0.479, F_4, 40_ = 0.838, *p* < 0.05) compared with the CRS model group (Figure 5B). It illustrated that FG treatment could reverse the spontaneity, non-spatial learning, and memory deficit induced by CRS.

### 3.3 Effects of fresh *Gastrodia elata* Blume on the Morris water maze test in chronic restraint stress-treated mice

**FIGURE 6:**
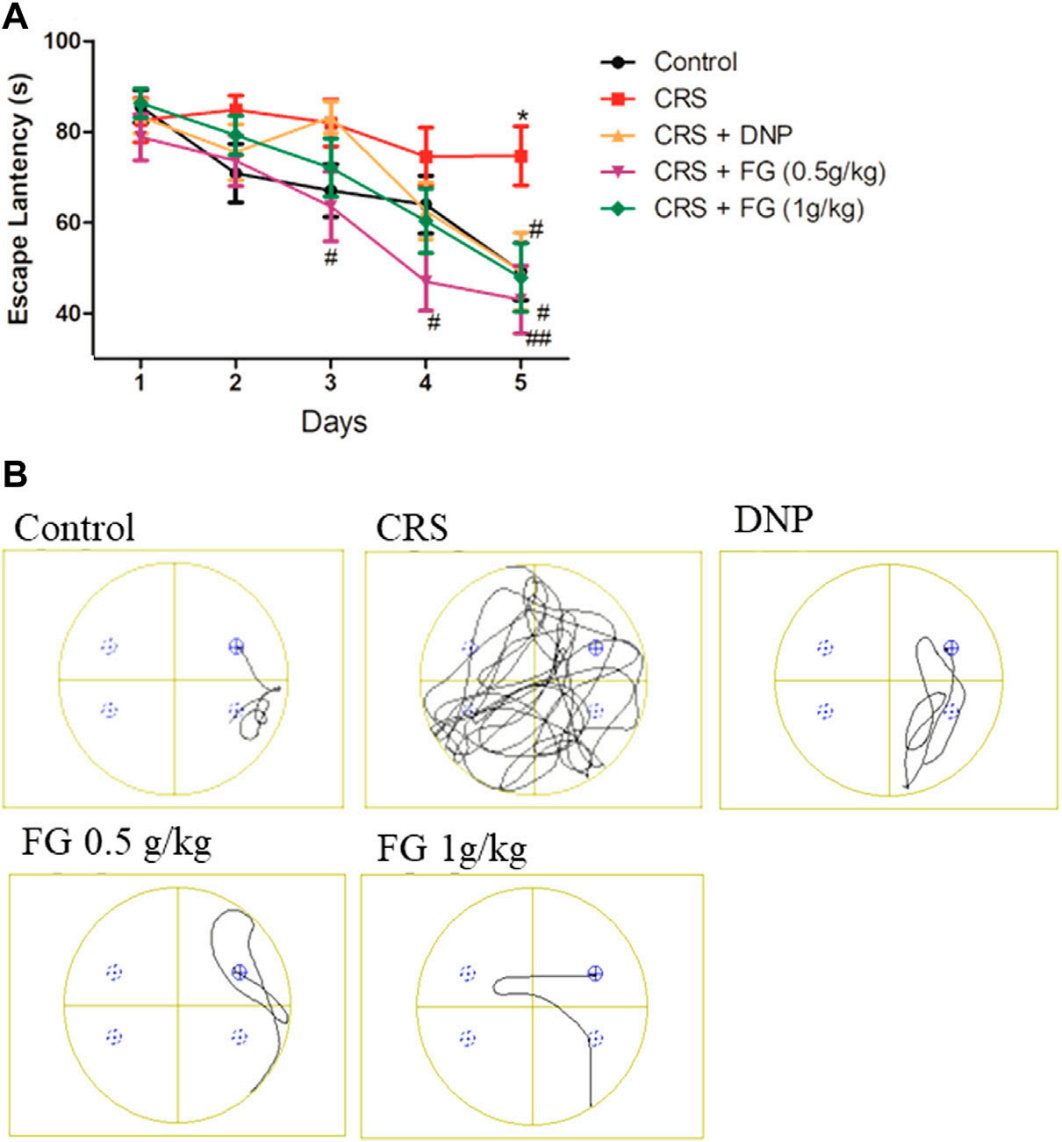
Effect of CRS, FG, and DNP on the MWMT of mice induced by CRS. Values represent the mean ± S.E.M. *n* = 9 ∼ 12 mice/group. **(A)** Escape latency; **(B)** Swimming track example (*p < 0.05 compared with the control group; ^#^ *p* < 0.05, ^##^ *p* < 0.01 compared with the CRS group.

MWMT is used for detecting the effects of FG on the long‐term, punitive spatial memory performance of CRS-treated mice. We first used two-way repeated measures ANOVA to analyze the interaction effects between the groups and days of training. The statistical analysis results revealed that the group had a significant effect on the escape latency (F_16, 416_ = 1.18, *p* < 0.001) in the navigation phase. On day 5 (Figure 6), compared with the control group, the CRS model group displayed a significantly longer escape latency (*p* < 0.05) and compared with the CRS model group, FG (0.5 and 1 g/kg) and DNP group could significantly shorten the escape latency (*p* < 0.01, *p* < 0.05). It is worth mentioning that FG low does (0.5 g/kg) group significantly attenuated the prolong escape latency induced by CRS from day 3 to day 5 (*p* < 0.05, *p* < 0.01). These indicated that CRS could induce long-term spatial memory impairment, and FG treatment can reverse it.

### 3.4 Effect of fresh *Gastrodia elata* Blume on the passive avoidance test

**FIGURE 7:**
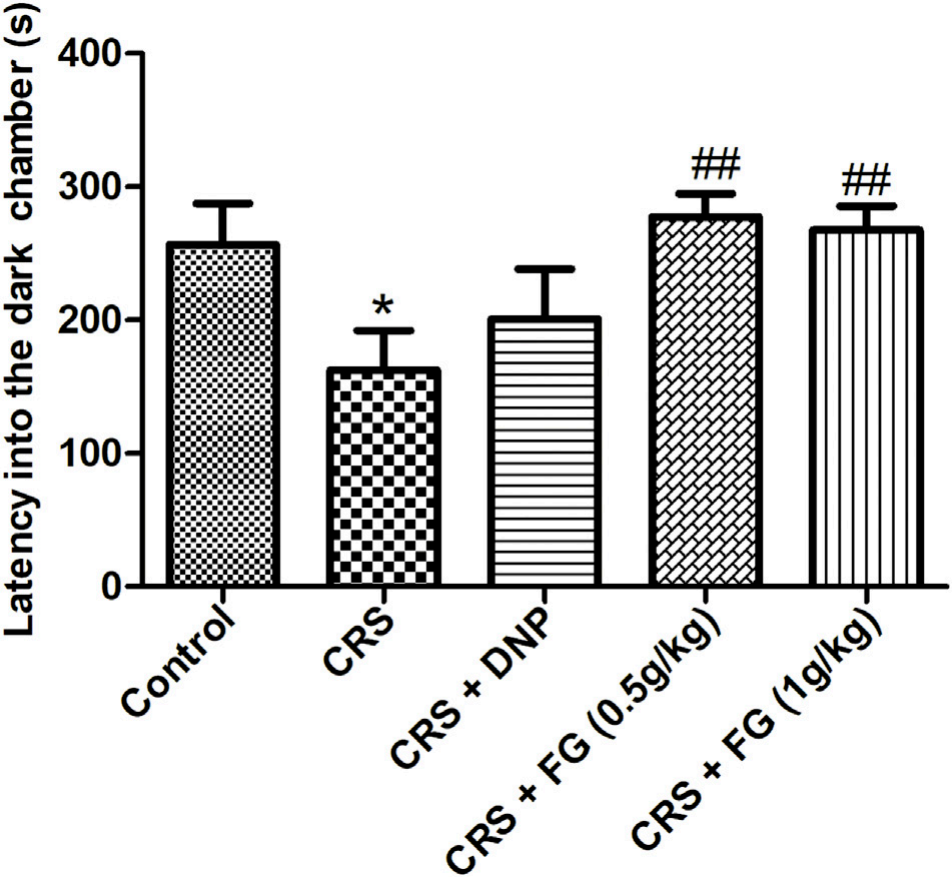
Effect of CRS, FG, and DNP on the PAT of mice induced by CRS. Values represent the mean ± S.E.M. *n* = 8 ∼ 10 mice/group. ^*^ *p* < 0.05 compared with the control group; ^##^ *p* < 0.01 compared with the CRS group.

As shown in Figure 7, the latency into the dark chamber of the CRS model group mice was significantly shorter than the control group (F_4,42_ = 0.328, *p* < 0.05). While compared with the model group, FG (0.5 and 1 g/kg) treatment effectively extended the latency into the dark chamber (F_4,42_ = 3.024, F_4,42_ = 2.094, *p* < 0.01).

### 3.5 Effect of fresh *Gastrodia elata* Blume on inflammatory response

**FIGURE 8:**
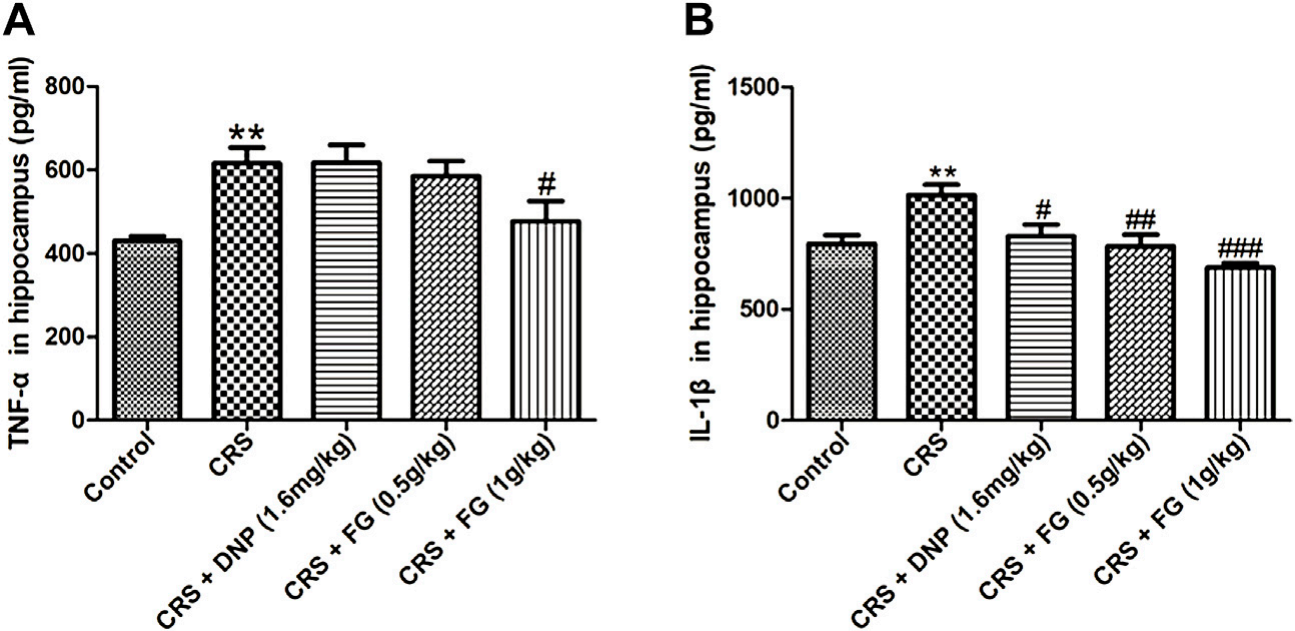
Effects of CRS, FG, and DNP on inflammation in serum of CRS-treated mice. **(A)** TNF-α and **(B)** IL-1β in serum. Values represent the mean ± S.E.M. *n* = 6 ∼ 8 mice/group. ^**^ *p* < 0.01 compared with the control group; ^#^ *p* < 0.05, ^###^ *p* < 0.001 compared with the CRS group.

To observe the CRS-induced inflammatory response in the hippocampus, we measured the expression of TNF-α and IL-1β. As shown in Figure 8, the CRS model group increased the TNF‐α and IL-1β levels in the serum compared with the control group with significance (F_4,34_ = 3.847, F_4,35_ = 0.153, *p <* 0.01). However, compared with the CRS group, FG administrated at 1 g/kg significantly inhibited the increases of TNF-α (F_4,34_ = 0.488, *p* < 0.05), FG (0.5 and 1 g/kg) and DNP markedly diminished the level of IL-1β (F_4,35_ = 0.150, F_4,35_ = 2.986, F_4,35_ = 0.437, *p* < 0.01, *p* < 0.001 and *p* < 0.05).

### 3.6 Effects of fresh *Gastrodia elata* Blume on the expression levels of molecules in the hippocampus

**FIGURE 9:**
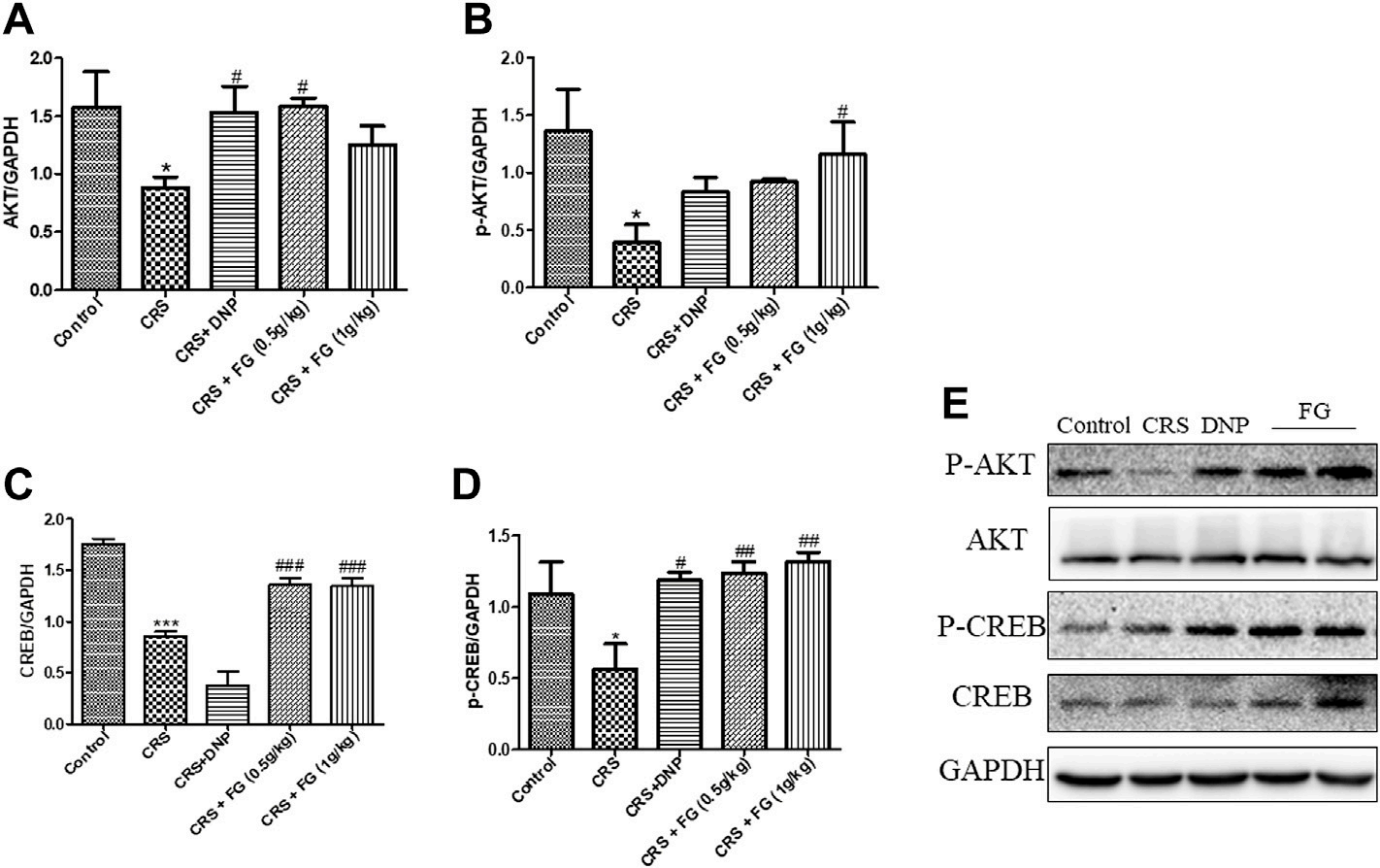
Effects of CRS, FG, and DNP on the AKT/CREB pathway expressed in the hippocampus of CRS-treated mice. **(A–E)** Expression levels of the protein were measured by western blot analysis and their respective histogram. *n* = 3 mice/group. Values represent the mean ± S.E.M. ^*^ *p* < 0.05, ^***^ *p* < 0.01 compared with the control group; ^#^ *p* < 0.05, ^##^ *p* < 0.01, ^###^ *p* < 0.001 compared with the CRS group.

**FIGURE 10:**
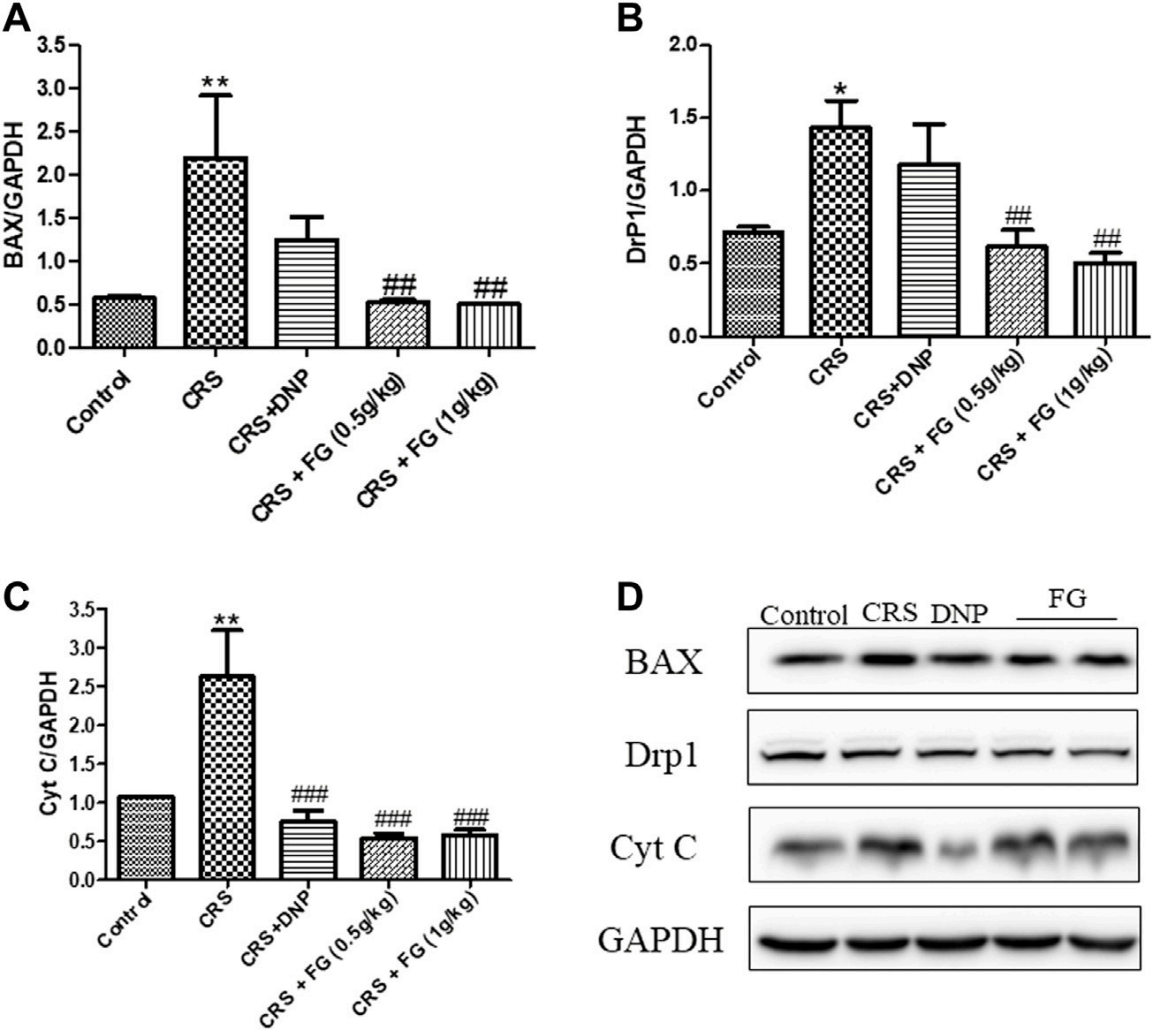
Effects of CRS, FG, and DNP on apoptosis-related proteins expressed in the hippocampus of CRS-treated mice. **(A–D)** Expression levels of the protein were measured by western blot analysis and their respective histogram. n = 3 mice/group. Values represent the mean ± S.E.M. ^*^ *p* < 0.05, ^**^ *p* < 0.01 compared with the control group; ^##^ *p* < 0.01, ^###^ *p* < 0.001 compared with the CRS group.

According to the results of the western blotting analysis (Figures 9, 10), the level of AKT, p-AKT, CREB, and p-CREB of the CRS group were lower than the control group (F_4,10_ = 4.721, F_4,10_ = 2.160, F_4,10_ = 3.832, F_4,10_ = 5.039, *p* < 0.05, *p* < 0.05, *p* < 0.001, *p* < 0.05), and the expression of Cyt C, DrP1, and BAX in the hippocampus of CRS-induced mice were significantly higher than the control group (F_4,10_ = 5.54, F_4,10_ = 4.164, F_4,10_ = 10.863, *p* < 0.01, *p* < 0.05 and *p* < 0.01, respectively). The administration of FG significantly reverse these changes (*p* < 0.05, *p* < 0.01, *p* < 0.001).

## 4 Discussion

The chronic restraint stress (CRS) model can simulate the physiological and psychological stress caused by long-term space-constrained environment and induce learning and memory impairment. In addition to studying the effects of general stress on human, CRS also plays an important role in investigating the effects of special environments such as aviation and sailing on human physical and mental health (Salehi et al., 2018; Salehpour et al., 2019; Peay et al., 2020). Also, CRS results in neuroinflammation, synaptic morphology alterations and mitochondrial apoptosis (Haack et al., 2008; Pietrogrande et al., 2018; Shen et al., 2021). Our research showed that the CRS model in mice could lead to cognitive impairment in the spontaneous and punishment behavioral test. FG was able to act against CRS‐induced memory impairment in mice and suggested that its action may be *via* anti-inflammatory and mediated mitochondrial apoptosis-related protein expression.

Animal behavior experiment is a subject that conducts observation and experiment in nature or in laboratory by collecting, observing, analyzing, and processing various behavior information of animals, which can be applied to study the physiological and pathological significance, as well as the generation mechanism of animal behavior expression (Sara, 2001; Xu et al., 2016b; Lu et al., 2018c; Lv et al., 2021). We applied a variety of animal behavior tests on learning and memory to assess the effect of FG. OLRT and NORT are sensitive, short-term, spontaneous activity behavioral methods to test the learning and memory ability of rodents through animals’ natural instinct of approaching and exploring novel object (Akkerman et al., 2012; Antunes and Biala, 2012). They were widely used in the study of memory neurobiology, brain injury mechanisms, and protective measures against cognitive impairment (Ennaceur, 2010; Xiong et al., 2020). The results showed that FG could reverse CRS-induced decrease in the DI, which implied that FG can ameliorate short-term spatial and non-spatial learning and memory. MWMT and PAT are punitive behavioral tests for learning and memory. Based on the rodents’ aversion of water, MWMT is widely used to test spatial memory (Vorhees and Williams, 2006). PAT is based on the dark preference habit of rodents, and mainly tests animals’ learning and memory ability to distinguish between light and dark (Zhang et al., 2018). In our results, FG significantly shortened the escape latency of animals in MWMT and prolonged the incubation period of mice to enter the dark room in PAT, indicating that the administration of FG can enhance the punitive long-term and short-term memory of mice. These animal learning and memory behavior results all together showed that FG could reverse various cognitive deficits induced by CRS.

Cellular activity in the brain depends on the high energetic support provided by the mitochondria (Hebert-Chatelain et al., 2016), enhancing the protein homeostasis of which can promote memory (Sorrentino et al., 2017). Inflammatory mediators regulate mitochondrial fission protein dynamin-related protein 1 (Drp1) (Yu et al., 2020), and the former can be induced to be released by CRS (24). Drp1 overexpression further accelerates the division of mitochondria and induces apoptosis (Shen et al., 2014), resulting in the release of Cyt c from the mitochondria into the cytoplasm (Tan et al., 2018), and meanwhile BAX plays a crucial role in the execution of apoptosis in the mitochondria (Rahmani et al., 2018). AKT directly protects the mitochondria from oxidants and its downstream substrate is cAMP response element binding (CREB) protein (Miyamoto et al., 2008), which is considered to increase the expression of synaptic plasticity protein, memory related protein, and new synapse formation. It is a key protein that affects memory storage and plays an important regulatory role in neuronal regeneration, synapse formation, learning, and memory (Villeda et al., 2014). Insulin and insulin growth factor can activate AKT through downstream signals, phosphorylate CREB, and increase the expression of synaptic plasticity genes and related proteins to promote synaptic neogenesis (Aguiar et al., 2011). It was previously reported that *Gastrodia elata* Blume can inhibit mitochondrial fragmentation and dysregulation of mitochondrial fusion and fission molecules (Huang et al., 2019). Therefore, we investigated whether the mechanism of FG reversing CRS-induced cognitive deficits is related to the control of mitochondrial-related protein. The results indicated that the FG can reduce the expression level of IL-1β and TNF-α significantly, which is consistent with the previous report (Xie et al., 2021). FG could significantly reverse CRS-induced mitochondrial fragmentation and dysregulation of mitochondrial fusion and fission molecule (Drp1 and Cyt C), and downregulate the level of BAX. We also observed that the levels of AKT, p-AKT, CREB, and p-CREB in the mice hippocampus were inhibited after CRS. Meanwhile, FG treatment dramatically reversed the decrease of AKT, p-AKT, CREB, and p-CREB protein expression in the hippocampus induced by CRS.

As mentioned previously, FG had a good effect on decreasing the level of TNF-α and IL-1β and promoting the expression of the AKT/CREB pathway and inhibition expression of Cyt C, DrP1, and BAX in the hippocampus. These may relate to a certain amount of gastrodin and p-hydroxybenzyl alcohol contained in FG (shown in Figure 1), which can reduce inflammation and inhibit division and apoptosis in the mitochondria (Jiao et al., 2019; Lei et al., 2020; Ma et al., 2020).

## 5 Conclusion

This study is the first to study the effects of FG on CRS-induced memory deficiency through both animal behavior tests and mitochondrial apoptosis-related protein detection. We found that the administration of FG exhibited an improvement effect in spontaneous short-term spatial and non-spatial memory (OLRT and NORT), as well as punitive non-spatial memory (PAT) and long-term spatial memory (MWMT). The enhancement of FG on learning and memory may be attributed to its mitochondrial apoptosis antagonism and anti-inflammatory effects. Thus, FG is considered to be a promising candidate supplement for ameliorating narrow space-induced hypomnesia, and this study provided a way for applying fresh *Gastrodia elata* Blume .

## Data Availability

The original contributions presented in the study are included in the article/Supplementary Material. Further inquiries can be directed to the corresponding authors.
